# Immune reactivity and host modulatory roles of two novel *Haemonchus contortus* cathepsin B-like proteases

**DOI:** 10.1186/s13071-021-05010-y

**Published:** 2021-11-19

**Authors:** Mariam Bakshi, Wenbin Tuo, Raffi V. Aroian, Dante Zarlenga

**Affiliations:** 1 Animal Parasitic Diseases Laboratory, USDA-ARS, Beltsville, MD USA; 2 Oak Ridge Institute for Science and Education, Oak Ridge, Tennessee USA; 3 University of Massachusetts Medical School, University of Massachusetts, Worcester, MA USA

**Keywords:** *Haemonchus contortus*, Gastrointestinal, Cathepsin B, Cysteine proteases, Peripheral blood mononuclear cells

## Abstract

**Background:**

*Haemonchus contortus* is a blood-feeding, gastrointestinal nematode (GIN) that causes significant economic losses to the small ruminant industry worldwide. Despite extensive efforts, our understanding of the molecular mechanisms used by GIN to evade host immune responses is limited. Cathepsin B-like proteins (CBPs) are members of the cysteine protease family and are involved in parasite invasion and thus provide viable vaccine candidates.

**Methods:**

In silico comparative analysis was used to identify conserved proteins among a subset of clade V parasitic nematodes with emphasis on blood-feeding worms, among which CBPs appeared prominently. We identified and characterized two novel CBPs designated Hc-CBP-1 and Hc-CBP-2. Rabbit anti-recombinant (r) Hc-CBP-1 and rHc-CBP-2 were used to detect the presence of native proteins in the excretory secretory products (ESP) and in worm tissues of adult *H. contortus*. Peptide arrays of rHc-CBP-1 and rHc-CBP-2 were screened with the homologous and heterologous anti-sera and with sera from dexamethasone-treated (Dex^+^) and non-treated (Dex^−^) *H. contortus*-infected animals to identify key immunogenic peptides. Gene transcription of Hc-*cbp-1* and Hc-*cbp-2* was also performed on *H. contortus*-infected animals treated with Dex^+^. Finally, the mature recombinant proteins were used to assess their abilities to modulate cell functions.

**Results:**

Immunohistochemistry showed that both Hc-CBP-1 and Hc-CBP-2 are present on the brush borders of the intestine; Hc-CBP-2 was also present in the hypodermis of the body wall. Peptide displays screened with rabbit anti-rHc-CBP-1 and anti-rHc-CBP-2 revealed regions within the proteins where dominant and overlapping epitopes prevailed. ELISA results were consistent with only Hc-CBP-1 being present in *H. contortus* adult ESPs. *H. contortus* from Dex^+^ animals exhibited a threefold increase in Hc-*cbp-2* transcript while Hc-*cbp-1* expression did not change. In contrast, comparisons of immunoreactivities of rHc-CBP-1 and rHc-CBP-2 peptide arrays to sera from Dex^+^ and Dex^−^ animals primarily showed changes in Hc-CBP-1 binding. Lastly, rHc-CBP-1 suppressed mRNA expression of bovine peripheral blood mononuclear cell cytokines/activation markers, including TNFα, IL-1, IL-6 and CD86.

**Conclusions:**

These results suggest that as secreted and cryptic proteins, respectively, Hc-CBP-1 and Hc-CBP-2 influence cellular and immunological activities that have interesting dynamics during infection and may provide viable immune-related targets for attenuating *H. contortus* infectivity.

**Graphic Abstract:**

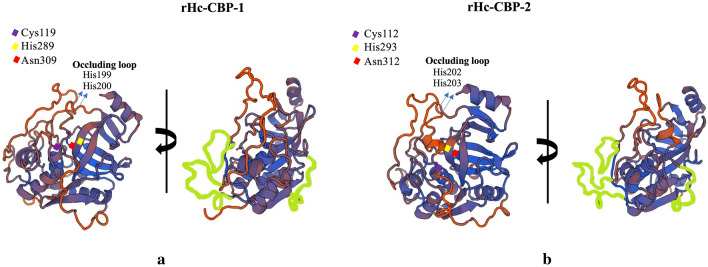

**Supplementary Information:**

The online version contains supplementary material available at 10.1186/s13071-021-05010-y.

## Background

Gastrointestinal nematode (GIN) infections are ranked among the most economically important diseases of livestock worldwide [1]. *Haemonchus contortus* is the most pathogenic of this group in small ruminants because both fourth-stage larvae (L4) and mature adult worms feed from capillaries in the abomasum. Infections are often accompanied by hemorrhagic gastritis, hypoproteinemia, anemia and edema, where acute infections can cause death [2]. A single adult worm is estimated to cause 30–50 μl of blood loss per day [3]. Overuse and abuse of anthelmintics have resulted in resistance to all major drug classes [4]. This, in conjunction with chemical residues in animal products, has advanced the need for alternative and sustainable control methods. Vaccination remains a viable albeit difficult option because it targets both resistant and susceptible strains of the parasite.

The design of effective vaccines is underpinned by advancing our understanding of host-parasite interactions, the immune responses involved in protection and the biology of the worm. While in the abomasum, parasite excretory secretory products (ESPs) are released that interact with the host to facilitate the infection process and modulate host immune responses [5–7]. Helminth infection can be accompanied by a disruption of the abomasa mucosa and hyperplasia of numerous cell lineages including epithelial cells [8], mast cells [9], lymphocytes [10–12] and eosinophils [13, 14]. To date, both *H. contortus* ESP (*Hc*-ESP) and digestive enzyme complexes have been tested for vaccine potential [15–19]. Some of these antigens constitute the native Barbervax™ vaccine, which is based upon a cryptic, gut-derived antigen preparation, H-Gal-GP, which consists of pepsins, metalloproteases, cysteine protease-like enzymes and H11, an integral membrane glycoprotein complex [20–22]. This vaccine is commercially available but sold to a limited market in Australia, New Zealand and South Africa because it is naturally derived from experimentally infected animals which constrains production and distribution. Unfortunately, protection is transient, requiring revaccination every 6 weeks. Recombinant DNA technology is one way to circumvent production issues, but this has been largely unsuccessful thus far [23].

The hematophagous lifestyle of *H. contortus* adult worms has armed them with an array of molecules essential for feeding such as cathepsin B-like proteases that digest hemoglobin, serum proteins and host cells [24]. Cathepsin B family members have been described in *H. contortus* that are important in pathogenesis; however, embryogenesis, molting, egg hatching and yolk degradation are additional functions associated with the protein family members making them viable targets for vaccine development [25, 26]. In other parasites like *Fasciola hepatica*, cathepsin B interferes with host immunity linked to peripheral blood mononuclear cells (PBMC) and induces pro-inflammatory responses [27]. During acute infections, cathepsin B from *Schistosoma mansoni* stimulates mixed Th1/Th2/Th17 immune reactions in mice resulting in a transient Th17 response [28]. Collectively, these and other data are consistent with an association between considerable immunogenic capacity and cathepsin B-like proteases.

Amidst extensive efforts to identify vaccine targets, studies are needed to better characterize deeper structural and immune-modulatory capacities imposed by *H. contortus* cathepsin B-like proteases and their roles as virulence factors. In the present study, two *H. contortus* cathepsin B-like cysteine proteases (CBP), Hc-CBP-1 and Hc-CBP-2, were identified using in silico analysis of select parasitic clade V nematodes and then biochemically characterized. Peptide assays were used to investigate immunogenic regions of recombinant (r) Hc-CBP-1 and rHc-CBP-2. The roles of these proteins in modulating host PBMCs, cytokine production and co-stimulatory surface marker expression were examined. We suggest that rHc-CBP-1 and rHc-CBP-2 are effective immunogens and hold promise as vaccine candidates against haemonchosis.

## Methods

### Identification of Hc-CBP-1 and Hc-CBP-2 by *in silico* analyses

Amino acid sequences for proteases and protease inhibitors from parasitic clade V nematodes [*H. contortus* (Additional file 1: Dataset S1)*, Teladorsagia circumcincta, Necator americanus, Ancylostoma caninum*)], non-parasitic nematodes (*Caenorhabditis elegans* and *Pristionchus pacificus*) and domestic sheep, *Ovis aries,* were obtained from NCBI and MEROPS databases. Putative secreted proteases were identified by the presence of amino-terminal signal sequences as predicated by SignalP 5.0 (http://www.cbs.dtu.dk/services/SignalP) algorithms. Family classification of protein sequences was determined from the conserved domain database in NCBI (CDD) [29].

Sequences from each species were subjected to local BLASTp (CLC Genomics Workbench, Qiagen, Hilden, Germany) with a BLOSUM62 matrix and an expectation value of 10.0. Sequences exhibiting > 60% similarity among *H. contortus, C. elegans, P. pacificus* and *O. aries* were excluded from further analysis. All other sequences from the *T. circumcincta*, *N. americanus* and *A. caninum* databases were aligned in pairwise fashion with the *H. contortus* database using local BLAST. This resulted in a subset of aspartate and cysteine proteases that were common among all databases. From the list of cysteine proteases, two *H. contortus* cathepsin B-like proteases, designated as Hc-*cbp*-1 and Hc-*cbp*-2 for gene and Hc-CBP-1 and Hc-CBP-2 for protein, were selected for further analysis.

### Parasite collection

All experimental protocols involving animals were approved by the Beltsville Animal Care and Use Committee and performed in accordance with relevant guidelines and regulations. Male Suffolk sheep and Holstein calves, 3–6 months of age, were inoculated with ~ 5000 and ~ 50,000 *H. contortus* infective L3, respectively, which were obtained by culturing feces, then purified by floatation in a lymphocyte separation medium and washed in 1× phosphate buffer saline (PBS), or from feces containing eggs that were cultured for 2 weeks in a moistened mixture of vermiculite at 25 ºC and then filtered twice through a Baermann funnel [30]. For adult worms, calves were killed at 21 days post infection (dpi). Adults were purified from abomasal contents by migration from agar slabs as described [30] and then washed three times with 1× PBS containing 1× penicillin/streptomycin and glutamine supplement. To collect *H. contortus* excretory secretory products (Hc-ESP), sheep were killed no less than 30 days post infection; 95% of adult worms were recovered, washed and cultured for 24 h in 1× Dulbecco’s modified essential medium (DMEM) (Lonza, Basel, Switzerland) supplemented with glutamine, 1× penicillin/streptomycin and gentamicin as described [31]. The culture supernatant was concentrated using Amicon spin columns (Millipore Sigma, Bedford, MA) and Pierce™ protein concentrators (Life Technologies, Carlsbad, CA). The purified Hc-ESPs were separated by electrophoresis on an 8–16% SDS-PAGE gel (Genscript, Piscataway, NJ) under reducing conditions and visualized by Coomassie blue stain. The remaining adult worms were fixed in 10% neutral formalin and used for immunohistochemical staining.

### Dexamethasone drug treatment in *H. contortus*-infected sheep

Six Suffolk sheep were maintained in a parasite-free environment with continuous feed and water according to an approved animal care protocol. Two groups of three animals were infected with 5000 *H. contortus* L3. One group (Dex^+^) was administered 10 ml of 2 mg/ml dexamethasone in the hind quarters on 21, 25, 29, 32 and 35 dpi [32]. The control group (Dex^−^) was given a similar volume of phosphate-buffered saline. On days 37, 38 and 39, animals were killed; heparinized blood and adult worms were collected. Serum was separated from the blood for ELISA, and adult worms were collected for transcript expression of Hc-*cbp-1* and Hc-*cbp-2* by quantitative PCR as described below.

### Structural analysis

The 3D structures of Hc-CBP-1 and Hc-CBP-2 were modeled in silico with the SWISS-MODEL protein structure homology modeling server [33]. The best template for each 3D structure was selected using the SWISS-MODEL template library search. The optimality of the predicted structures was estimated using global model quality estimation (GMQE) and qualitative model energy analysis (QMEAN) [34] where GMQE combines the properties from the target-template alignment and the template search methods. The obtained structures were validated using Ramachandran plots [35]. The structures with a higher number of residues in favored regions (> 90%) and fewer residues in outlier regions were selected.

### Recombinant protein expression of Hc-CBP-1 and Hc-CBP-2

Total RNA was isolated from *H. contortus* adult worms homogenized in TRIzol® reagent (Thermo Fisher Scientific, Carlsbad, CA). Crude RNA was DNAse treated, column purified (Zymo Research, Irvine, CA) and eluted with nuclease-free water. Template cDNA was synthesized using an cDNA synthesis kit as recommended by the manufacturer (Thermo Fisher Scientific, Carlsbad, CA). The cDNAs were PCR amplified using forward and reverse primers with restriction sites, EcoRI and NotI, for Hc-CBP-1 and SalI and NotI for Hc-CBP-2 (Table 1), and the amplified products were inserted into the pCR2.1 cloning vector (Thermo Fisher Scientific, Carlsbad, CA) by TA cloning. Isolated plasmids were restriction enzyme digested and the inserts subcloned into the expression vector pET29b ( +) containing a hexa-histidine tag for downstream protein purification. Plasmids containing subcloned fragments were used to transform *E. coli* BL21 for protein expression, and the reading frames of the final products were validated by automated sequencing.

**Table 1:** Hc-*cbp-1* and Hc-*cbp-2* recombinant protein expression and qPCR primers

Gene name	Primer sequence Bold and underlined: restriction sites	Recombinant protein expression (E) or qPCR (Q) primers
Hc-CBP-1	F:**GAATTC**TACGGAAGTACTTGGGGATTCTA R:**GCGGCCGC**TAGTCTTCTAGTCACGTCGTT	E
Hc-CBP-2	F: **GTCGAC**CAAGCGGATGTGCTCGCTGCTT R: **GCGGCCGC**CCCTTGAACATGCCCGGCGA	E
Hc-*cbp-1*	F: ACAAGTTCTACGGCAAGGGA; R: GCGGTGTGTTTGTAGACTCC	Q
Hc-*cbp-2*	F: GCGAGGACGCTTACGAATTA; R: GGAGAAGTCCTCGTAAACAGTG	Q

Following sequence verification, overnight cultures of each clone were transferred to 500 ml LB containing 50 µg/ml kanamycin and then grown at 37 °C until the OD_600_ = 0.5–0.7. Isopropyl β-d-1-thiogalactopyranoside was added to a final concentration of 0.5 mM to induce protein expression, and the incubations were continued at 16 °C for 20 h. The cells were collected by centrifugation at 10,000×*g* for 10 min and the pellets sonicated in lysis buffer (50 mM NaH_2_PO_4,_ pH 7.4; 300 mM NaCl, and 10 mM imidazole). The lysates were centrifuged at 12,000×*g* for 20 min and the cleared supernatants passed over Ni–NTA agarose affinity columns (Macherey–Nagel, Düren, Germany) and eluted in 5-ml increments with elution buffers containing imidazole gradient (10 mM–400 mM). The purified fractions were analyzed in SurePAGE^TM^ (Genscript, Piscataway, NJ) 4–20% BIS-TRIS gradient gel and stained by Coomassie stain. Samples containing the highest amount of recombinant protein in the absence of non-specific binding were pooled and concentrated. Protein concentrations were determined using a bicinchoninic acid (BSA) quantification kit (Thermo Fisher Scientific, Carlsbad, CA). Rabbit polyclonal antibodies against purified rHc-CBP-1 and rHc-CBP-2 were generated by Thermo Fisher Scientific (Carlsbad, CA) and validated by Western blot analysis. Prior to downstream studies, endotoxin was removed from rHc-CBP-1 and rHc-CBP-2 preparations using Triton X-114 [31] with final concentration of 1% and then vortexed for 10 s. The homogeneous mixture was then incubated on ice for 5 min, warmed to 37 °C for 10 min to promote phase separation and then spun for 10 min. The upper aqueous layer was transferred to a fresh tube, and the process was repeated. Endotoxin levels were evaluated using an endotoxin detection kit (Limulus Amoebocyte Lysate Kit, Qiagen, Hilden, Germany).

### Detection of native Hc-CBP-1 and Hc-CBP-2 in *H. contortus* adults and Hc-ESPs

Adult crude worm extracts and Hc-ESPs were quantified and diluted to 1 µg in 100 µl in ELISA coating buffer (sodium bicarbonate buffer, pH 9.0) and then loaded onto 96-well plates for overnight incubation at 4 °C. The plates were blocked with 0.05% Tween-20 and 1× PBS (PBS-Tween) containing 5% bovine serum albumin (BSA) and then incubated with polyclonal antibodies against rHc-CBP-1 or rHc-CBP-2 (1:200) in 1× PBS containing 5% BSA. After washing, the wells were incubated with horseradish peroxidase (HRP) conjugated goat anti-rabbit secondary antibody (1:1000) (Thermo Fisher Scientific, Carlsbad, CA) for 1 h and then washed with PBS-Tween-20. Substrate (ABTS; 2,2’-azino-bis (3-ethylbenzothiazoline-6-sulfonic acid) was added, and the reaction was stopped using 1% sodium dodecyl sulfate (SDS). Plates were read at 410 nm using a SpectraMax spectrophotometer (Molecular Devices, San Jose, CA). All samples were assayed in triplicate.

### Immunolocalization of native Hc-CBP-1 and Hc-CBP-2 in *H. contortus* adult worms

*Haemonchus contortus* adult worms were collected from the abomasa of infected animals, fixed in 10% neutral formalin, embedded in paraffin and sectioned at 5 µm thickness (HistoServe Inc., Germantown, MD, USA). The slides were deparaffinized and rehydrated through xylene/ethanol washes, then quenched with 3% H_2_O_2_ and rehydrated prior to antigen recovery in 0.4% pepsin 1% hydrochloric acid at 37 °C for 15 min. The sections were washed twice with 0.75% of 30% BRIJ-35 (Millipore Sigma, Burlington, MA) in 1× PBS (BRIJ–PBS), blocked with 0.5% sodium caseinate in BRIJ–PBS for 10 min and then incubated with rabbit antisera to rHc-CBP-1 or rHc-CBP-2 (1:800 dilution) and pre-immune sera (1:800 dilution) for 30 min at room temperature. After washing, the slides were incubated with goat anti-rabbit HRP conjugated secondary antibody (GARP) for 20 min and treated with 3,3' diaminobenzidine (DAB) substrate (Abcam, Cambridge, MA). Hematoxylin and HistoMark TrueBlue (VWR, Radnor, PA) were used as counterstains. Micrographs were generated using the Zeiss Axioskiope 2 Plus microscope (Zeiss, Thornwood, NY, USA).

### Quantitative PCR of Hc-*cbp-1* and Hc-*cbp-2* in *H. contortus* derived from Dex^+^ and Dex^−^ injected animals

Five *H. contortus* adult worms were hand-selected from each animal, homogenized in TRIzol and then used for total RNA isolation as per the manufacturer’s instructions. Quantitative PCR (qPCR) was performed in triplicate from synthesized cDNA (1:10 dilution) in 25 µl volume containing 300 nM each (Table 1) and 2× SYBR green PCR master mix (Bio-Rad, Hercules, CA) as follows: 50 °C for 2 min, 95 °C for 10 min, then 40 cycles of 95 °C for 15 s and 60 °C for 1 min. Primer efficiencies were tested using melting curve analysis (Bio-Rad, Hercules, CA). Hc-*cbp-1*- and Hc-*cbp-2*-specific qPCR primers were selected and validated from aligned sequences and generated fragments of 121 bp and 104 bp, respectively. The mRNA expression levels for Hc-*cbp-1* and Hc-*cbp-2* were determined using the ΔΔCt method [36]. Glyceraldehyde 3-phosphate dehydrogenase (GAPDH) was used as a housekeeping gene for normalization [37].

### Hc-CBP-1 and Hc-CBP-2 peptide arrays

Forty-six peptides, 15 amino acids (aa) in length, were synthesized (New England Peptide, Garner, MA) with an 8-aa overlap among adjacent peptides (Additional file 6: Dataset S6) and collectively spanned the lengths of each mature protein [Hc-CBP-1 (329 aa) and Hc-CBP-2 (328 aa)]. Peptides encompassing putative signal peptide regions were not included. Each peptide was reconstituted in 200 µl dimethyl formamide. Each peptide (10 µl) in a total volume of 200 µl coating buffer was plated in duplicate and incubated overnight at 4 °C. Peptides were incubated overnight at 4 °C with rabbit polyclonal antibodies to rHc-CBP-1-, rHc-CBP-2- (1:1000) and *H. contortus*-infected sheep sera (1:1000). Sheep sera (1:250) from *H. contortus*-infected and Dex^+^- and Dex^−^-treated sheep were also incubated overnight at 4 °C with each peptide array separately. All wells were washed with 1× PBS-Tween and then incubated with either goat anti-rabbit HRP conjugated secondary antibody (1:1000) or goat anti-sheep IgG-HRP conjugated antibody (1:1000) at room temperature for 1 h. Colorimetric detection was performed using ABTS substrate after the addition of 1% SDS to terminate the reactions. Positive binding was visualized at 410 nm using a SpectraMax spectrophotometer (Molecular Devices, San Jose, CA). Each of these arrays was repeated twice and done in duplicate and triplicate.

### PBMC activation assay using rHc-CBP-1

Bovine blood was collected into sodium citrate buffer using Vacutainers® (Beckton Dickinson, Franklin Lakes, NJ). Cells (PBMC), isolated by density gradient centrifugation in Ficoll-Paque (GE healthcare, Chicago, IL), were washed with balanced salt solution (BSS; 5.5 mM anhydrous glucose, 0.05 mM CaCl_2_, 1.0 mM MgCl_2_, 5.4 mM KCl, 145 mM TRIS, and 140 mM NaCl, pH 7.4) then stained with trypan blue to assess viability. Purified PBMCs (5 × 10^4^) were seeded onto 24-well tissue culture plates in 1 ml DMEM and treated with 0.1, 1 or 10 μg/ml of endotoxin-free rHc-CBP-1 or PBS (control). Native Hc-CBP-1 was not observed in Hc-ESP and therefore was not tested. The cathepsin B inhibitor CA-074 (Millipore Sigma., Burlington, MA) was used to suppress cathepsin B protein activity in the presence of 0.1, 1 or 10 μg/ml endotoxin-free rHc-CBP-1. Lipopolysaccharide (LPS) (kindly provided by Dr. Harry Dawson, USDA, Beltsville, MD) and Concanavalin A (Con A) were used separately as positive controls. The culture plates were incubated at 37 °C with 5% CO_2_ for 72 h. The cells were collected for total RNA isolation and cDNA synthesis as described above. Quantitative PCR (qPCR) was performed in triplicate from cDNA (1:10 dilution) in 25 µl containing 300 nM each of primer [IL-1, IL-6, tumor necrosis factor (TNF)α, CD40, CD80 and CD86] [30] and 2× SYBR green PCR master mix (Bio-Rad, Hercules, CA) as follows: 50 °C for 2 min, 95 °C for 10 min, then 40 cycles of 95 °C for 15 s and 60 °C for 1 min. Cognate mRNA expression levels were determined using the ΔΔCt method [36] using GAPDH as a housekeeping gene for normalization [37]. Primer efficiencies were validated using melting curve analysis (Bio-Rad, Hercules, CA).

### Statistical analysis

Quantitative PCR results were normalized to expression values obtained from *H. contortus* adults from Dex^−^ animals. One-way analysis of variance followed by Tukey’s and/or Dunnett’s post hoc test was done to compare pre-immune and immunized animals and relative fold changes in transcription between Dex^+^ and Dex^−^ animals. Two-tailed pairwise *t*-test was done to compare peptide arrays screened with sera from Dex^+^ and Dex^−^ animals. The antibody-binding signal intensities between animals were normalized to mean absorbance signal intensities of each group across the entire array using the formula: absorbance/mean absorbance of two animals × 100. The data are represented as means ± standard error (SE) where *p* ≤ 0.05 is considered statistically significant using Prism 8.4.1 (Graph Pad Software Inc, San Diego, CA).

## Results

### *In silico* discovery and 3D modeling of Hc-CBP-1 and Hc-CBP-2

**Fig. 1:**
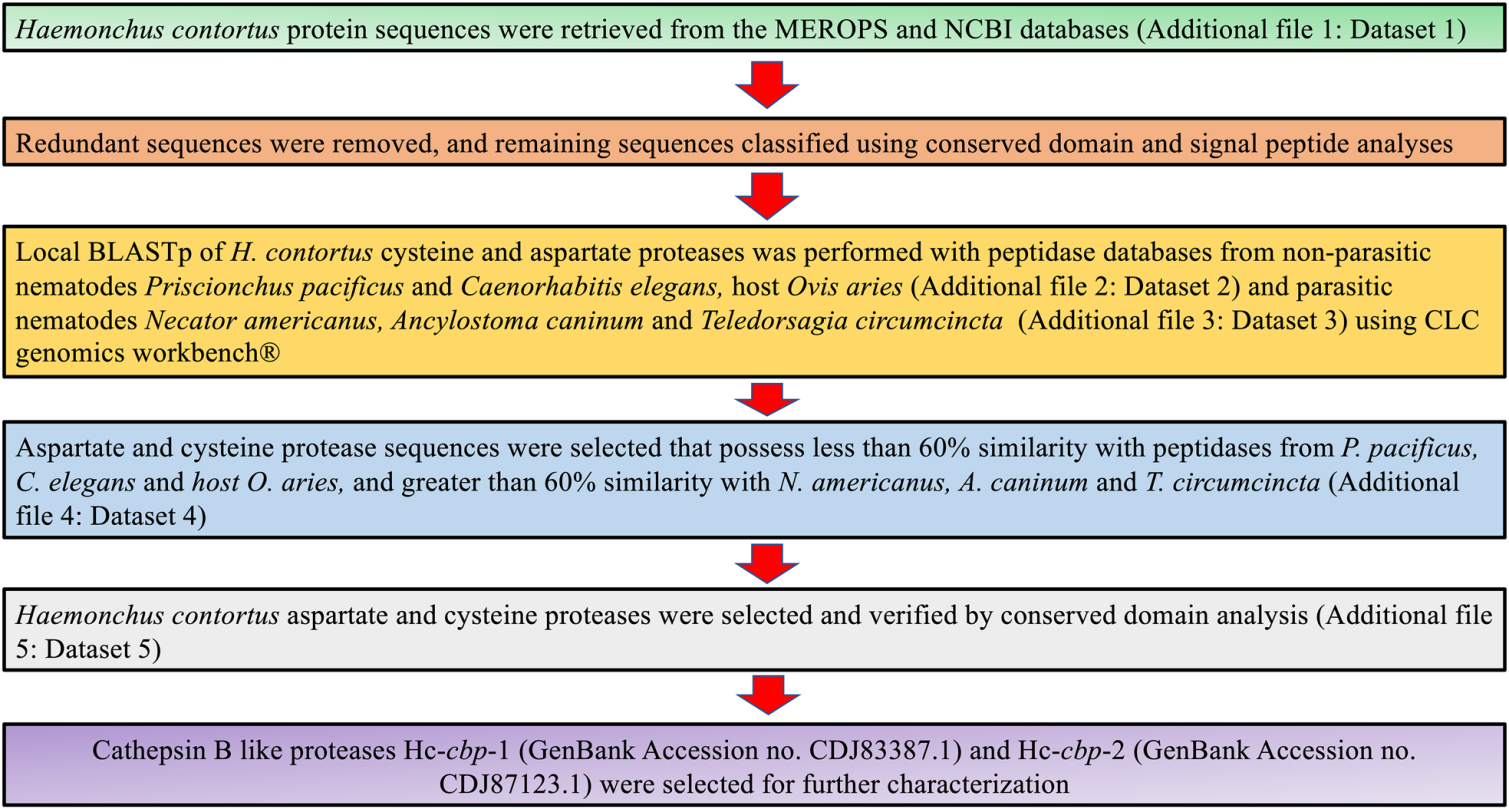
In silico analysis for identifying *H. contortus* cathepsin B-like proteases, Hc-CBP-1 and Hc-CBP-2. *H. contortus* proteases were selected from NCBI and MEROPS databases and subjected to local BLASTp against clade V parasitic helminths

Using the culling mechanisms described in Fig. 1, *H. contortus* sequences that showed < 60% identity with *O. aries*, *P. pacificus* and *C. elegans* were selected (Additional file 2: Dataset S2). From this list, 114, 106 and 145 sequences from *H. contortus* exhibited > 60% identity with sequences from *T. circumcincta*, *A. caninum* and *N. americanus*, respectively (Additional file 3: Dataset S3). Among these, 69 proteases were present in all four species (Additional file 3: Dataset S3 and Additional file 4: Dataset S4); two were defined as cathepsin B-like cysteine proteases (Additional file 4: Dataset S4 and Additional file 5: Dataset S5) and designated Hc-*cbp-1* (GenBank accession no. CDJ83387.1) and Hc-*cbp*-*2* (GenBank accession no. CDJ87123.1). The predicted rHc-CBP-1 and rHc-CBP-2 protein sequences were 349 and 346 amino acids in length with theoretical molecular masses of 39.56 and 38.57 kDa, respectively.

**Fig. 2:**
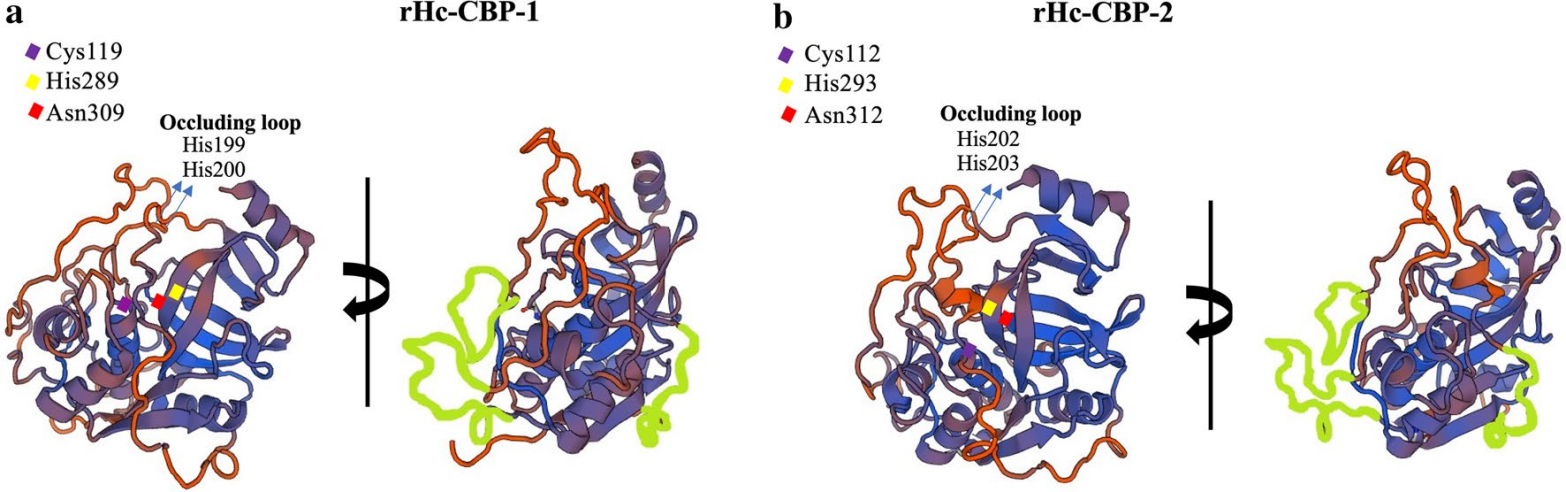
Three-dimensional modeling of *H. contortus* cathepsin B-like proteases Hc-CBP-1 and Hc-CBP-2. The 3D models of Hc-CBP-1 and Hc-CBP-2 were generated using the x-ray crystallographic structure of mature cathepsin B from the genus *Rattus* using SWISS-MODEL. The catalytic triad residues Cys119 (**a**) and Cys112 (**b**) were in the left domain and His289 (**a**) and His293 (**b**) and Asn309 (**a**) and Asn312 (**b**) in the right domain. On the right panel, the surface-exposed loops specific to *H. contortus* are highlighted in green. The residues delimitating the loops—Leu73-Pro80, Asn182-Pro193, Arg221-Asp233 for Hc-CBP-1 and between Arg-74-Asp89, Tyr186-Pro194 and Cys221-Thr238 for Hc-CBP-2—are highlighted in green using Microsoft Paint (Microsoft Corporation, Redmond, WA)

The putative structures of native Hc-CBP-1 and Hc-CBP-2 were determined using the SWISS-MODEL [36] and the catalytic domain of the mature Rat cathepsin B, which shares 46% and 43% sequence identity with rHc-CBP-1and rHc-CBP-2, respectively. Both rHc-CBP-1 and rHc-CBP-2 exhibit the typical papain-like fold that is characteristic of members of the cathepsin family (Clan CA, family C1) and are composed of left (L) and right (R) domains [38] (Fig. 2). The left domain has three helical regions, and the right domain is composed of a barrel of six strands, which includes a shorter α–helical motif where the catalytic triad is housed in a cleft separating the two domains. The 3D models of rHc-CBP-1 and rHc-CBP-2 show that the two domains containing Cys119 (Fig. 2a) and Cys112 (Fig. 2b) are in the left domain, and His289 (Fig. 2a), His293 (Fig. 2b), Asn309 (Fig. 2a) and Asn312 (Fig. 2b) are in the right domain. Both structures exhibit the canonical occluding loop between the conserved Cys197 and Asp214 residues in Hc-CBP-1 (Fig. 2a) and the Cys200 and Asp215 in Hc-CBP-2 (Fig. 2b) and are further characterized by two adjacent histidine residues (His199 and His200 for rHc-CBP-1 and His202 and His203 for rHc-CBP-2) that putatively block the active site cleft responsible for the dipeptidyl carboxypeptidase activity. The major structural differences in backbone superposition correspond to additional residues and are localize to the surface-exposed loops, which include residues Leu73-Pro80, Asn182-Pro193 and Arg221-Asp233 for Hc-CBP-1 and Arg74-Asp89, Tyr186-Pro194 and Cys221-Thr238 for Hc-CBP-2.

**Fig. 3:**
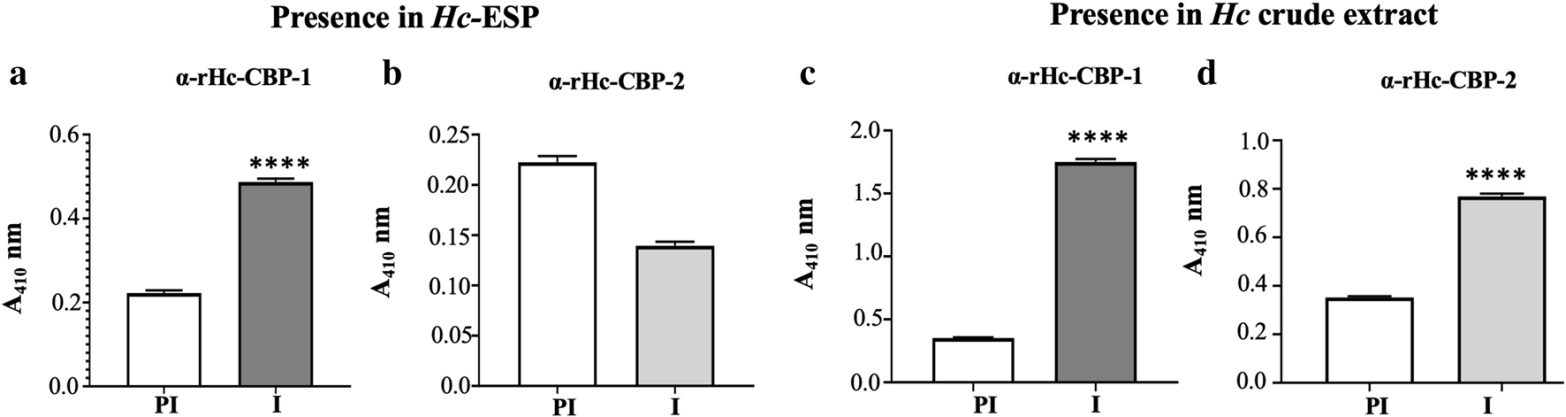
Detection of native Hc-CBP-1 and Hc-CBP-2 in adult Hc-ESP and crude worm extract. ELISA was used to detect Hc-CBP-1 and Hc-CBP-2 in Hc-ESP (**a**) and adult crude worm extract (**b**) using anti α-rHc-CBP-1 (**a** and **c**), α-rHc-CBP-2 (**b** and **d**) and pre-immune serum (1:200 dilution). Data are reported as the mean (M) of triplicates ± SEM captured at Absorbance of 410 nm (A_410_). ****p ≤ 0.0001 indicates statistical significance between pre-immune (PI) and immune (I) serum samples. NS means not significant

### Expression of rHc-CBP-1 and rHc-CBP-2 and detection of native proteins

The full-length cDNA sequences encoding recombinant (r) rHc-CBP-1 and rHc-CBP-2 are identical to those in the GenBank database where expression was achieved in bacterial expression system (Additional file 7: Figure S1) which were used to generate polyclonal antibodies. Using polyclonal rabbit antibodies to rHc-CBP-1 and rHC-CBP-2, the native Hc-CBP-1 was found to be highly expressed in the Hc-ESP (Fig. 3a), whereas Hc-CBP-2 was undetectable in the Hc-ESP by ELISA (Fig. 3b). However, both Hc-CBP-1 (Fig. 3c) and Hc-CBP-2 (Fig. 3d) were present at high levels in crude extracts of adult worm. Immune reactivity of the rHc-CBP-1 antiserum to adult worm extracts was significantly stronger than with Hc-CBP-2 antiserum (Fig. 3c, d).

**Fig. 4:**
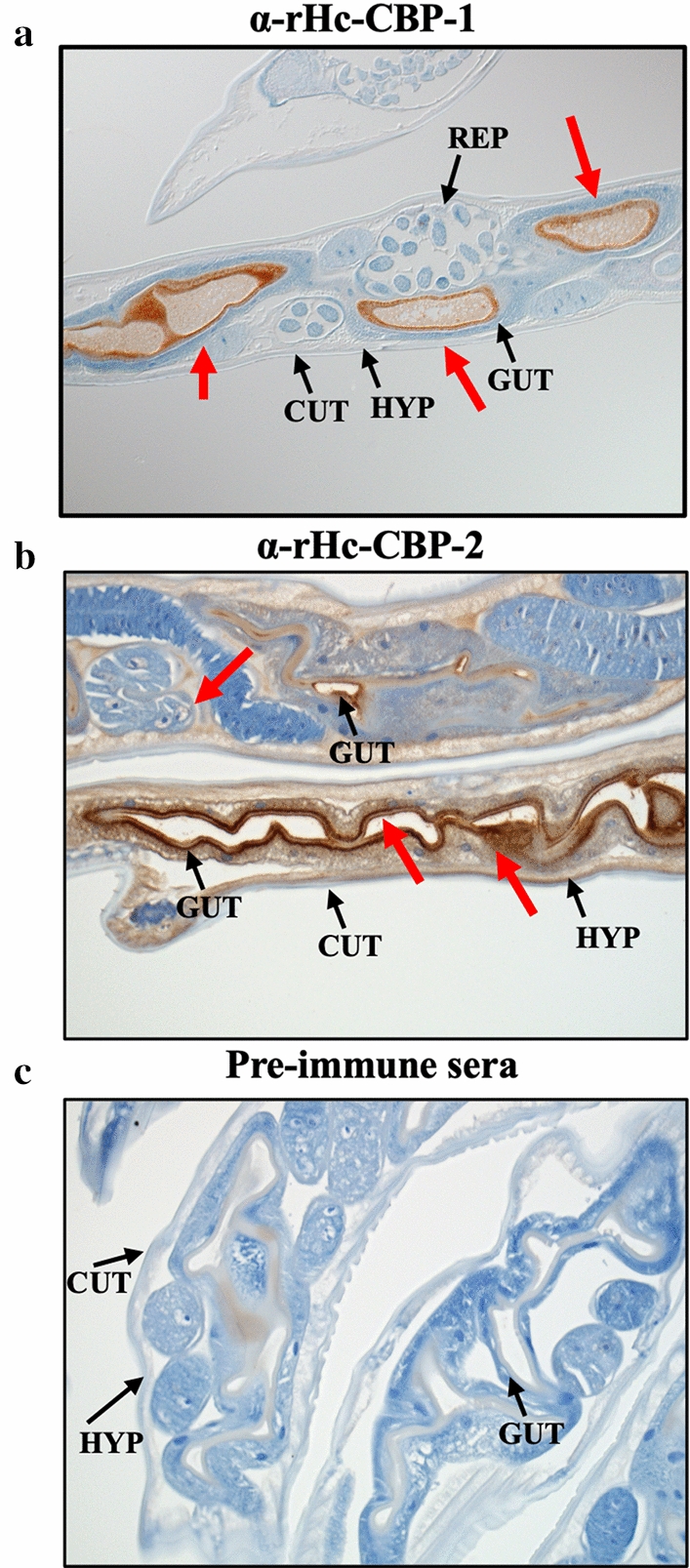
Immunolocalization of native Hc-CBP-1 and Hc-CBP-2 in *H. contortus* adults. Immunohistochemical staining of sectioned *H. contortus* adult worms was performed using rabbit anti-α-rHc-CBP-1 (**a**), α-rHc-CBP-2 (**b**) antibody and pre-immune serum (**c**) (1:800 dilution). CUT = cuticle, GUT = gut, REP = reproductive organs, HYP = hypodermis. Magnification = 10× (**a**); 20× (**b**, **c**). Red arrows indicate the positive binding of α-rHc-CBP-1 or α-rHc-CBP-2 antibody to the tissues

Immunohistochemical staining using rabbit polyclonal antibodies to each homologous protein localized native Hc-CBP-1 and Hc-CBP-2 to the brush borders or apical end of the intestinal epithelial cells (Fig. 4); however, anti-rHc-CBP-2 bound more intensely with the terminal web below the microvilli and to the hypodermis along the intestinal epithelial cell layer (Fig. 4b). No staining was observed in the adult worms when pre-immune rabbit serum was used (Fig. 4c).

### Overlapping peptide binding patterns of rHc-CBP-1 and rHc-CBP-2

**Fig. 5:**
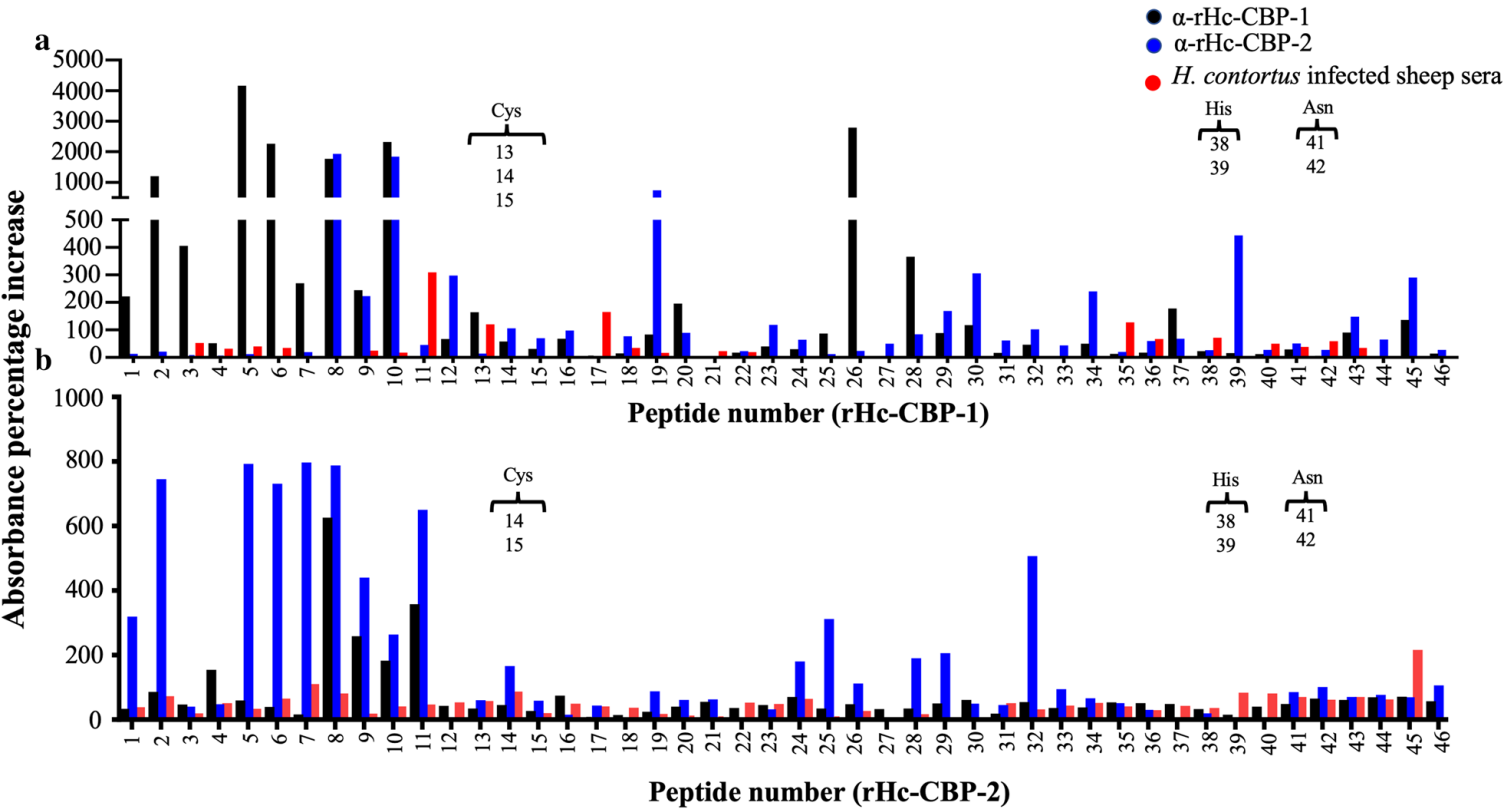
Peptide arrays of Hc-CBP-1 and Hc-CBP-2 using homologous and heterologous antisera. Forty-six 15-amino-acid peptide with 8 amino acid overlaps among adjacent peptides were generated for Hc-CBP-1 (**a**) and Hc-CBP-2 (**b**). Antibody binding to each peptide was evaluated by ELISA at 410 nm using rabbit α-rHc-CBP-1 and α-rHc-CBP-2, and sheep *H. contortus*-infected serum. Percent changes between immune and pre-immune sera (× 100) were plotted. Presence of active site is indicated in brackets. Cys = cysteine, His = histidine, Asn = aspargine. The data are represented as means ± standard error (SE); values where *p* ≤ 0.05 were considered statistically significant

Peptide displays of rHc-CBP-1 and rHc-CBP-2 were screened with the homologous and heterologous rabbit antibodies and with *H. contortus*-infected sheep sera (Fig. 5). Given that both proteins are members of the cathepsin family and share sequence identity, some cross-reactivity was observed when each peptide display was screened with the heterologous antibodies; however, distinct immune reactivity profiles were also observed between each array and the homologous antisera. Figure 5a shows that the rHc-CBP-1 antibodies bound most strongly to peptides 1, 2, 3, 5–10, 26 and 28 in the homologous array where peptides 8–10 were well recognized by antisera to both rHc-CBP-1 and rHc-CBP-2. rHc-CBP-1 peptides 19, 30, 34 and 39 showed strong binding to rabbit anti-rHc-CBP-2. In like manner, antiserum to rHc-CBP-2 bound most strongly to homologous rHc-CBP-2 peptides 1, 2, 5–11, 25, 28, 29 and 32 where rHc-CBP-2 peptides 8–11 cross-reacted with rabbit anti-rHc-CBP-1 (Fig. 5b). Serum from sheep infected with *H. contortus* reacted minimally with rHc-CBP-1 (peptides 11, 17 and 35) and rHc-CBP-2 (peptide 45).

### Transcriptional and immunological changes in Hc-*cbp-1* and Hc-*cbp-2* in immunosuppressed animals

**Fig. 6.**
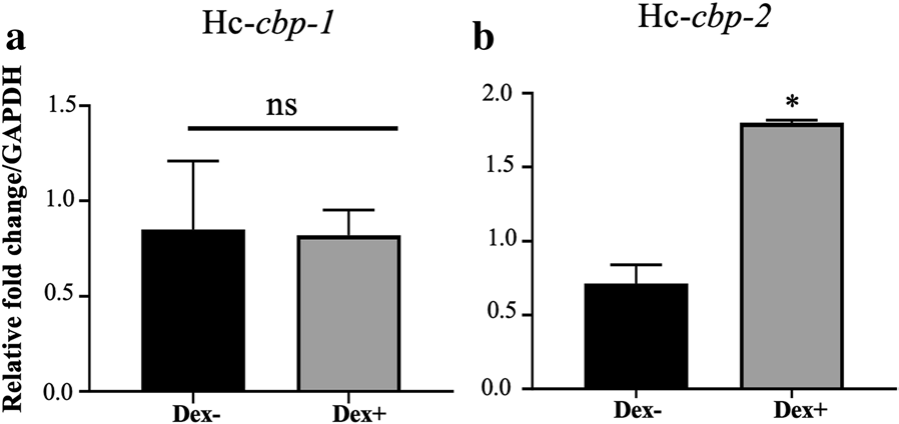
Transcript abundance of Hc*-cbp-1* and Hc-*cbp-2* in Dex^+^- and Dex^−^-treated *H. contortus*-infected sheep. Relative transcript abundance for Hc-*cbp-1* (**a**) and Hc-*cbp-2* (**b**) was determined using cDNA synthesized from adult *H. contortus* total RNA obtained from Dex^+^ and Dex^−^ (control) animals and Hc-*cbp-1* and Hc-*cbp-2 *specific qPCR primers. The values were normalized to adult *H. contortus* from Dex^−^ animals and GAPDH was used as internal control. Data were analyzed using the ΔΔCt method where *p ≤ 0.05. Data are reported as mean (M) of three replicates ± SEM

Transcript levels of Hc-*cbp-1* and Hc-*cbp-2* were evaluated in adult worms acquired from Dex^+^- and Dex^−^-treated animals (Fig. 6). As shown in Fig. 6a, the Hc-*cbp-1* transcript encoding the parasite secreted protein was not affected by Dex^+^ treatment of the host animals; however, the transcript level of Hc-*cbp-2*, where the cognate protein was not found in Hc-ESP, was nearly threefold higher in Dex^+^ treated animals (Fig. 6b).

**Fig. 7:**
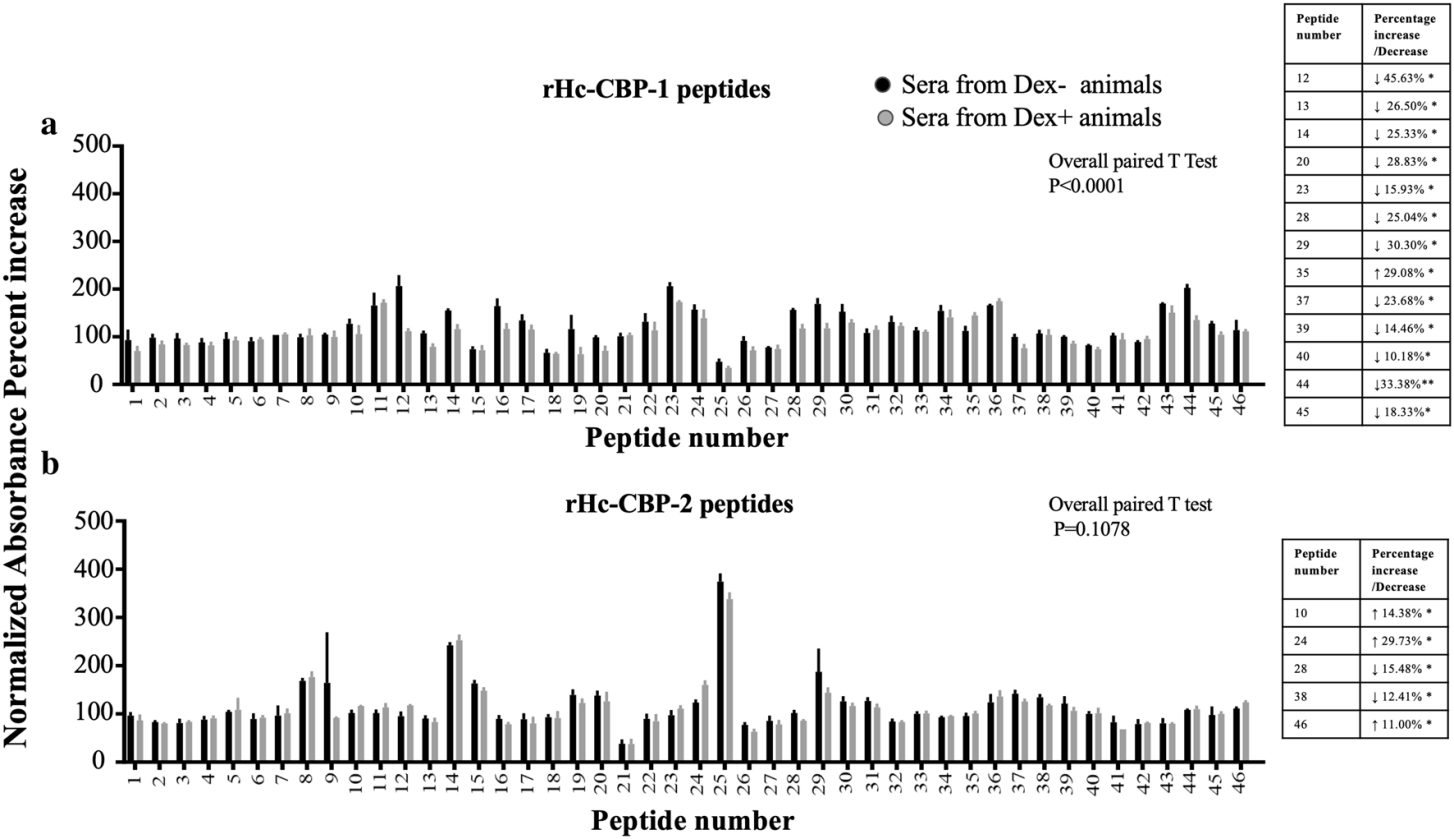
Peptide arrays of Hc-CBP-1 and Hc-CBP-2 using sera from Dex^+^ and Dex^−^ *H. contortus*-infected sheep. Hc-CBP-1 (**a**) or Hc-CBP-2 (**b**) specific peptides (1-46) were incubated with serum obtained from Dex^+^ and Dex^−^ animals. Percent change between drug treated and non-drug treated (×100) were plotted. The antibody binding percent signal intensities between animals were normalized by averaging the absorbance intensities of two animals/group using the formula: absorbance/mean absorbance of two animals × 100 ± standard error (SE); values where *p* ≤ 0.05 are considered statistically significant

As shown in Fig. 7, there were also differences in the overall binding of sera from Dex^+^-treated animals to the peptide arrays. Based upon a two-tailed, pairwise *t*-test, numerous peptides from rHc-CBP-1 exhibited lower binding affinity to Dex^+^ infected sera than to Dex^−^ sera (*p* < 0.0001) suggesting suppressed immunity to this secreted antigen (Fig. 7a). Specifically, peptides 12, 14, 16, 23, 28, 29, 30 and 37 showed a reduction in antibody titers compared to control animals. In contrast, peptides from the non-secreted Hc-CBP-2 did not show significant differences in antibody binding between Dex^+^ and Dex^−^ animals (*p* = 0.1078); only peptides 25 and 29 exhibited some variation in binding suggesting that the non-secreted Hc-CBP-2 is not affected by alterations in the host immune response (Fig. 7b).

### rHc-CBP-1 modulates co-stimulatory and pro-inflammatory cytokines

**Fig. 8:**
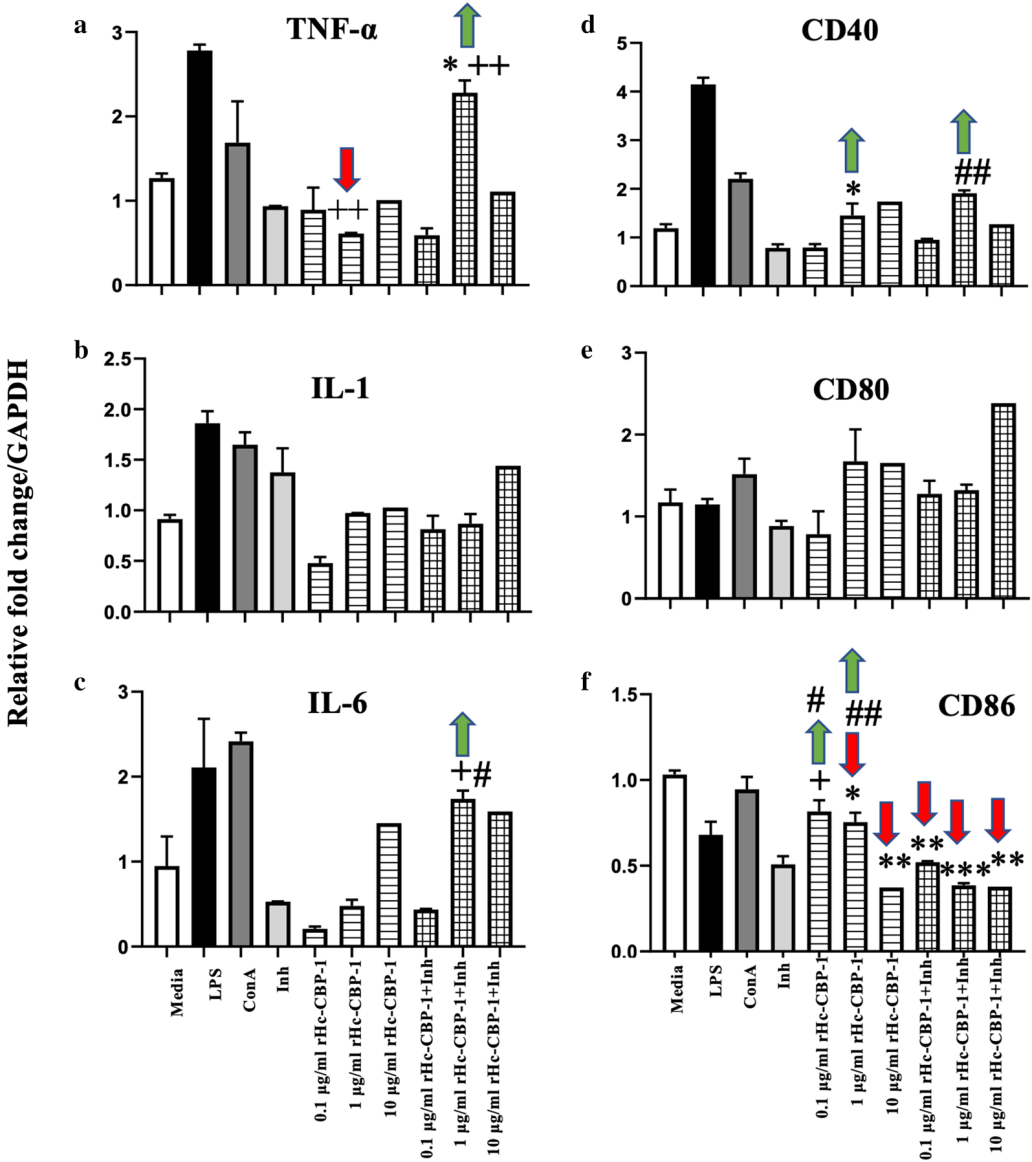
Activity of rHc-CBP-1 on bovine peripheral blood mononuclear cells. Relative transcript abundance of pro-inflammatory cytokines (TNFα (**a**), IL-1 (**b**), IL-6 (**c**)) and co-stimulatory markers (CD40 (**d**), CD80 (**e**) and CD86 (**f**)) were evaluated on bovine PBMCs following 72 h of incubation with 0.1, 1 and 10 μg/ml concentrations of rHc-CBP-1. Data are reported as the mean (M) of two biological and three technical replicates. Statistically significant differences between media controls vs samples, inhibitor vs mix of inhibitor and rHc-CBP-1 (0.1, 1 and 10 µg/ml) and rHc-CBP-1 (0.1, 1 and 10 µg/ml) vs mix of inhibitor and rHc-CBP-1 (0.1, 1 and 10 µg/ml) are defined as follows: (*), ( +), (#) = *p* ≤ 0.05; (**), (+ +), (##) = *p* ≤ 0.01; (***), (+ + +), (###) = *p* ≤ 0.001. The arrows indicate the direction of change in transcript abundance normalized to media only

Since Hc-CBP-1 is secreted, we examined the role of rHc-CBP-1 in modulating immune cells for transcriptional changes in three pro-inflammatory cytokines (TNFα, IL-1 and IL-6) and three cell surface markers (CD40, CD80 and CD86) resulting from treatment of PBMCs from bovine blood with endotoxin-free rHc-CBP-1 (Fig. 8). As shown in Fig. 8a, TNF-α transcription was significantly downregulated in the presence of 1 μg/ml rHc-CBP-1, the effects of which were reversed in the presence of cathepsin B inhibitor. This response appeared biphasic in that TNF-α expression did not change in the presence of 0.1 or 10 μg/ml rHc-CBP-1 (Fig. 8a). When the expression of IL-1 was examined (Fig. 8b), the data were inconclusive; rHc-CBP-1 seemed only to suppress mRNA levels at the lowest concentration (0.1 μg/ml) where, in the presence of inhibitor, suppression returned to normal levels. At the intermediate and highest concentrations tested (1.0–10 μg/ml rHc-CBP-1), mRNA values were unaffected except in the presence of inhibitor where mRNA expression values exceeded baseline levels; however, these responses were not statistically significant. When IL-6 was examined (Fig. 8c), transcription was downregulated at 0.1 and 1 μg/ml rHc-CBP-1 with a trend toward control levels at the highest concentration of rHc-CBP-1. These effects were altered in a statistically significant manner in the presence of cathepsin B inhibitor and became muted at higher concentrations of rHc-CBP-1 (10 μg/ml).

With respect to the expression of co-stimulatory markers, Fig. 8d indicates that CD40 mRNA levels increased in a dose-dependent manner when cells were incubated in the presence of rHc-CBP-1, though the presence of inhibitor seemed not to alter this trend. The rHc-CBP-1 also upregulated the expression of CD80 where these effects were most pronounced at higher concentrations of rHc-CBP-1 (1 μg/ml and 10 μg/ml) (Fig. 8e). As with CD40, these trends were not reversed in the presence of inhibitor. The effects of rHc-CBP-1 on CD86 expression (Fig. 8f) were consistent with a concentration-dependent downregulation of the marker. The effects of this downregulation were exacerbated rather than attenuated in the presence of inhibitor (Fig. 8f).

## Discussion

Cathepsin B-like proteases are major virulence factors for helminth parasites and thus attractive therapeutic targets. Nematode cysteine proteases are often expressed in glands or intestinal cells and can act as key "digestive enzymes" [25]. Several studies have shown that helminth cathepsin B-like proteases can modulate host-pathogen interactions [39–41]. Our primary interest was to examine a subset of cysteine proteases in *H. contortus* that are shared among a subset of other clade V nematode parasites, in particular, blood-feeding nematodes. An in silico analysis of key datasets identified a family of genes linked to cathepsin B-like proteases, two of which encode proteases that harbor the classical Cys, His and Asn catalytic triad, suggesting that native Hc-CBP-1 and Hc-CBP-2 function as endopeptidases.

In vitro proteolytic activity was not observed for either rHc-CBP using common host substrates, i.e. hemoglobin, IgGs or collagen (data not shown). Thus, native activities and substrates remain unresolved. It is possible that the proteins did not self-cleave to the active form in the presence of substrate. Alternatively, the lack of proper folding of the recombinant proteins and absence of post-translational modifications may also contribute to inactivity of rHc-CBP-1 and rHc-CBP-2. However, both functionality and substrate affinity of these cathepsin-like proteins is continuing using a mammalian expression system to generate the recombinant proteins.

Native Hc-CBP-1 and Hc-CBP-2 are both present within the brush borders of the intestinal wall of the *H. contortus* adult worms where only Hc-CBP-1 was also found in adult Hc-ESPs. Additionally, Hc-CBP-2 was detected underneath the cuticle possibly in the hypodermis of adult worms and in additional brush borders of the intestinal wall. It is possible that Hc-CBP-2 is secreted in other stages of the parasite; however, this was not examined. Adult *H. contortus* cysteine proteases have been linked to the digestion of host blood; however, these were defined as cryptic proteins and not found in Hc-ESP [20].

Cysteine proteases in helminths have been shown to digest hemoglobin under the acidic conditions present in the parasite gut and food vacuoles [42]. According to the present study, however, Hc-CBP-1 is not a cryptic protein but is actively secreted into the worm’s local environment and may help modulate at the host:parasite interface while in the abomasum where the L4 and adults feed. In contrast, amidst the presence of a secretory signal, Hc-CBP-2 was not secreted from the worm. However, caution must be taken when interpreting these data because active secretory signals define cellular events not parasitological activities. Namely, Hc-CBP-2 may indeed be secreted from the cell but not necessarily into the milieu that is transported outside the adult worm. Indeed, immunohistochemical staining showed Hc-CBP-2 was not confined to the parasite intestines, but was present throughout the hypodermis beneath the cuticle. This is consistent with regulating internal activities such as molting, cuticle remodeling and embryogenesis [26]. Also, Hc-CBP-2 may function as a cryptic parasite protein that interacts only with ingested host contents but remains attached to the worm’s intestinal wall. Thus, it is unlikely Hc-CBP-1 and Hc-CBP-2 represent redundancies in functionality, and they may therefore differ in their natural substrates.

Mutagenesis and pH studies have demonstrated that the occluding loop of cathepsin B is a hydrogen ion-dependent gate for controlling not only the type of activity (exo- or endo-) but also maturation to the active state [43]. At low pH where activation is optimal, the occluding loop is thought to displace the N-terminal region of the protein, making it available for autoproteolysis. Once activated, cathepsin B functions as an exopeptidase at low pH; however, cathepsin B proteases can also operate outside of the lysosomal fraction [44]. Hence, as demonstrated for other cathepsin B-like enzymes in *H. contortus*, Hc-CBP-1 and Hc-CBP-2 could function as both endo- and exopeptidases.

The use of peptide arrays screened with antisera can be effective for identifying immunodominant epitopes and developing subunit vaccines [45, 46]. Herein, we characterized and compared antibody epitopes among rHc-CBP-1 and rHc-CBP-2 using both homologous and heterologous rabbit polyclonal antibodies and sheep infection serum. The observed immunodominant peptides were relegated to the exposed N-terminal loop region of rHc-CBP-1 and rHc-CBP-2 and less so to the internally located active sites which are folded inward. In our analysis, we also found that peptides 8, 9, 10 and 11 bound to both homologous and heterologous antisera. Not surprisingly, the overall immune profiles generated using rabbit antisera to rHc-CBP-1 and rHc-CBP-2 and the sheep sera from experimentally infected animals were not congruent where rHc-CBP-1 and rHc-CBP-2 were poorly recognized by the sheep sera. Although this is predictable with the cryptic Hc-CBP-2, it was less so with the secreted Hc-CBP-1. Amidst a 57% sequence similarity among the two Hc-CBP, the peptide profiles for the homologous and heterologous rabbit antibodies were relatively distinct, further supporting a lack of redundancy in substrate activities among these cathepsins [47]. However, based upon these arrays, the few areas of overlap in antibody binding may still afford targets for the development of conserved subunit vaccines.

Dexamethasone is an immune-suppressive drug that is widely used to treat immune conditions in sickened animals; however, it has also been used to investigate host-parasite relationships [48–50]. Surprisingly, the transcript levels of the secreted Hc-*cbp-1* did not change in Dex^+^ animals whereas the non-secreted, Hc-*cbp-2* transcript increased significantly. This suggests that production and therefore secretion of Hc-CBP-1 is little effected by the immune status of the host and that the non-secreted Hc-CBP-2, likely a cryptic protein, must interact directly with ingested host contents because it appears downregulated or controlled by a healthy immune system. In agreement with the changes in transcription, host antibody levels to Hc-CBP-2, which is attached to the parasite intestinal wall, were slightly higher in Dex^+^ animals relative to controls, which is perplexing. Native Hc-CBP-2 does possess a secretory signal, and its secretion may be related to the immune status of the host, which could explain why transcription levels increased in immunocompromised animals. Others who have studied dexamethasone treatment in sheep infected with *Haemonchus* demonstrated overall lower antibody responses relative to non-treated animals [48].

*Haemonchus contortus* L4s and adults feed on blood for nutrients; however, host blood also contains immune factors that the parasite must circumvent to maintain blood flow and its own survival. Host PBMCs contain monocytes, B cells, T cells, dendritic cells and macrophages [51], the collection of which can induce Th1 responses that drive inflammation and Th2 responses that resolve inflammation. Typically, Th1 responses are characterized by changes in TNFα, INFγ, nitric oxide, IL-1 and IL-6, which are important in promoting and maintaining proinflammatory responses during infection. Inflammation-related responses can also be monitored by examining cell activation via cell surface markers. One such co-stimulatory marker, CD40, is a 50-kDa molecule expressed on different activated cell types, including monocyte macrophages [52]. Two other surface molecules, CD80 and CD86, are also important in immune cell activation in response to pathogens [53–55] and associated with inflammation. On this basis, our data suggest that the secreted rHc-CBP-1 downregulates TNFα, IL-1 and IL-6 transcripts in host PBMCs inhibiting the host Th1 environment. In contrast, we also found that rHc-CBP-1 induces significant expression of CD40 and suppression of CD86, indicating a mixed response. Recently, Chen et al. showed that recombinant cathepsin B from *Fasciola gigantica* induces PBMC cell cytokine profiles consistent with an active role in parasitism and host control [56]. Taken together, these data are consistent with Hc-CBP-1 immunomodulating the host inflammatory cells to advance its survival and growth. More detailed mechanisms involving key molecules of apoptosis, cell proliferation and signaling molecules merit further investigation.

In summary, we have described two *H. contortus*-derived cathepsin B-like proteases that show sequence similarity to proteins in other clade V parasite nematodes. Although both were selected based upon the presence of putative secretory signals, and both were found in the exposed layers of the intestinal epithelium, only Hc-CBP-1 was present in Hc-ESP of adult worms. Peptide arrays demonstrated regions comprising both specific and overlapping antibody binding between the two cathepsins. The greatest cross reactivity occurred predominantly within the N-terminal region of the mature proteins where N-linked glycosylation and the formation of an active protease originate. This suggests that pan-specific antibodies can be developed against this region that in turn may prevent the formation of an active protein. It is interesting to note that rabbit antibodies bound very strongly to the N-terminal region of both rHc-CBP-1 and rHc-CBP-2, which were largely unrecognized by sheep infection serum, suggesting that natural immunity is lacking. We also show that rHc-CBP-1 modulates pro- and anti-inflammatory responses within the myriad of cells that make up PBMCs and that responses to both these proteins are affected by the immune status of the host suggesting again that they are viable targets for immune intervention against *H. contortus.*

## Supplementary Information

**Additional file 1: Dataset S1. ***H. contortus* protein sequences retrieved from MEROPS and NCBI databases.

**Additional file 2: Dataset S2. ***H. contortus* cysteine and aspartate proteases that were less than 60% similar to peptidases from *P. pacificus*, *C. elegans* and *O. aries.*

**Additional file 3: Dataset S3. ***H. contortus* cysteine and aspartate proteases that were greater than 60% similar to peptidases from *T. circumcincta*, *A. caninum* and *N.* *americanus.*

**Additional file 4: Dataset S4. **Selected *H. contortus* cysteine and aspartate proteases that are in common with peptidases from *T. circumcincta*, *A. caninum *and *N. americanus.*

**Additional file 5: Dataset S5. **Conserved domain search of selected *H. contortus* cysteine and aspartate proteases.

**Additional file 6: Dataset S6. **Overlapping peptides derived from Hc-CBP-1 and Hc-CBP-2 protein sequences.

**Additional file 7: Figure S1. **Recombinant protein expression of Hc-CBP-1 and Hc-CBP-2. Recombinant proteins rHc-CBP-1 (A) and rHc-CBP-2 (B) were eluted in 15 mM to 400 mM imidazole, separated on SurePAGE^TM ^(Genscript, Piscataway, NJ) 4–20% BIS-TRIS gradient gel and then stained with Coomassie blue.

## Data Availability

Data available on request from the authors.
