# Supplementary figures and images for: Immune reactivity and host modulatory roles of two novel Haemonchus contortus cathepsin B-like proteases
Source: Parasit Vectors. 2021 Nov 19;14:580. doi: 10.1186/s13071-021-05010-y (PMC8603344; doi:10.1186/s13071-021-05010-y)

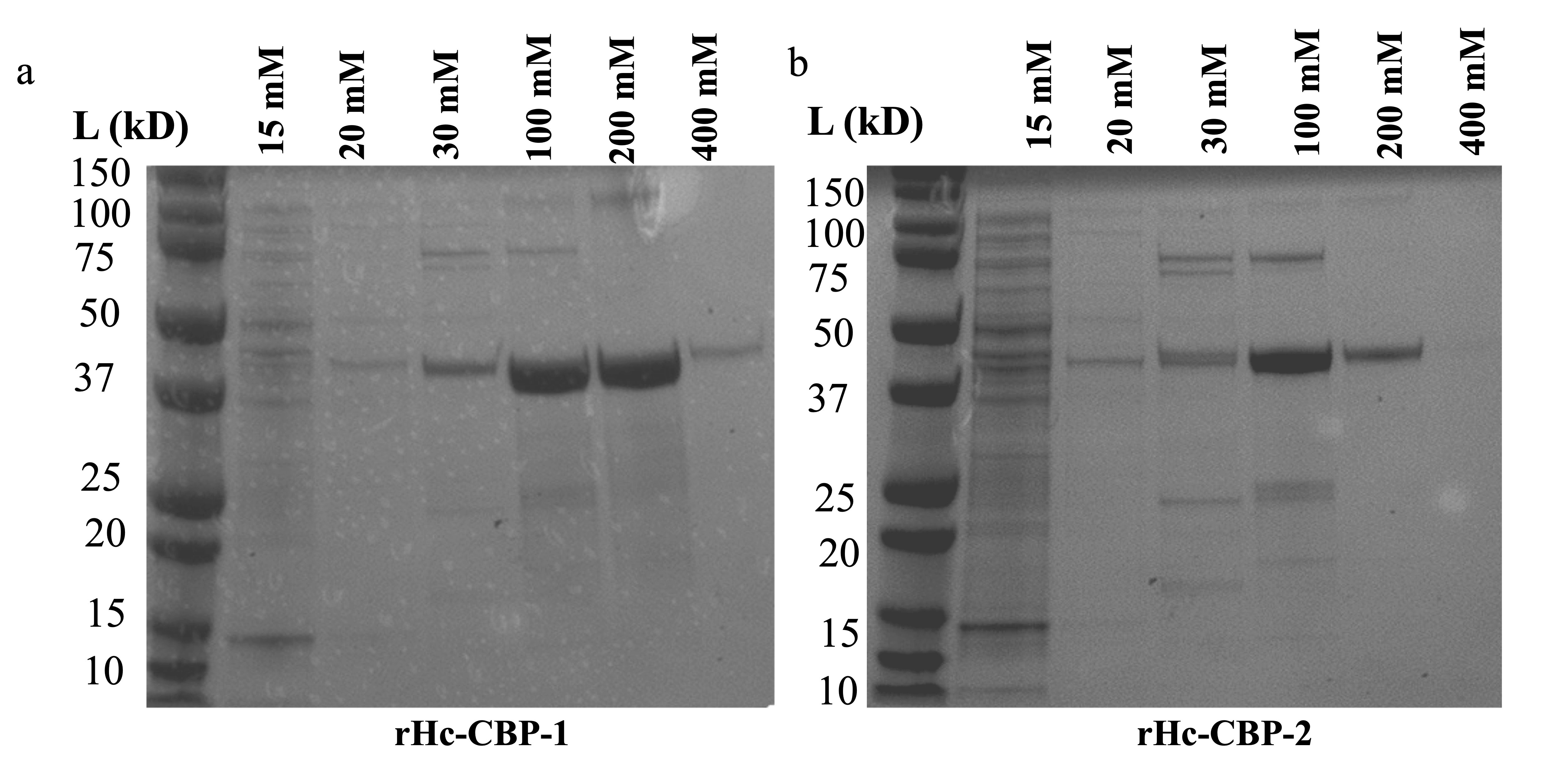

Supplement: Supplementary file 1 — Additional file 1: Dataset S1. H. contortus protein sequences retrieved from MEROPS and NCBI databases. [file 13071_2021_5010_MOESM1_ESM.png]
